# Estimating q−GEVL distribution parameters under Type II progressive censoring using particle swarm optimization

**DOI:** 10.1371/journal.pone.0323897

**Published:** 2025-05-28

**Authors:** Rasha Abd El-Wahab Attwa, Shimaa Wasfy Sadk, Hassan M. Aljohani

**Affiliations:** 1 Department of Mathematics and Statistics, College of Science, Zagazig University, Zagazig, Egypt; 2 Department of Mathematics and Statistics, College of Science, Taif University, Taif, Saudi Arabia; Covenant University, NIGERIA

## Abstract

In this article, the effect of the parameters in the properties of a well-known distribution called q-extended extreme value with linear normalization is discussed. Moreover, these parameters are estimated by both maximum likelihood and Bayesian approaches using type-II progressive censoring. The removals of type-II progressive censoring are considered under three well-known random removals (Fixed, discrete uniform, and binomial). Finding effective numerical techniques is a typical challenge for statisticians when estimating MLE parameters for distributions with many parameters. So one of our aims in this article is to show how the metaheuristic optimization like the particle swarm optimization, can handle this problem. Furthermore, the interval estimation for the parameters is calculated using the Fisher information matrix. The Bayesian approach is utilized for both the informative and non-informative under two different loss functions (square error and Linex loss functions) using Lindley’s approximation. Moreover, home price data in California represent a good fit for the q-extended extreme value distribution with linear normalization. By using this fitting some of California’s future home prices are predicted by using the return level function.

## 1 Introduction

Extreme value theory (EVT) is a very interesting essential branch of statistics for modeling the behavior of extreme events. EVT has many applications, such as insurance, cybersecurity, environmental science, and other fields. [1] provided a good insight into the importance of EVT in these areas. One of the most famous distributions on EVT is the generalized extreme value under linear normalization (GEVL) distribution. The GEVL distribution is considered a continuous probability distribution that models the maximum (or lowest) of a group of independent, identically distributed random variables. The GEVL distribution has three parameters: location, scale, and shape. These parameters determine tail heaviness and skewness, which are summarized by [2]. Many researchers used GEVL in their investigation as [3–5]. In statistical theory, new standard distributions are currently widely used. Usually, generators are used to add a new parameter and combine preexisting distributions to create a new family see [6–12]. The concept of *q*-analogs is used in many different kinds of mathematical and statistical contexts, giving a framework for generalizing classical concepts and structures by introducing a new parameter *q*. The use of *q*- analogs in probability and statistics enables the investigation of a wider range of distributions and features that can be reduced to their initial distribution when q→1. Here in this article, we considered the *q*-generalized extreme value under linear normalization (q−GEVL) distribution. The q−GEVL distribution is an extension of the GEVL distribution that incorporates a *q* parameter, providing greater flexibility for modeling as illustrated by [13]. Many other articles considered the *q*-analog of their work as [14–19].

In statistical reliability and survival analysis, censoring describes instances in which recognizing the failure of all units may be impossible due to time, cost, or other aspects. Sometimes, it occurs because the observation period expires before all events (such as failures or fatal crashes) have occurred. To handle this dilemma, the researcher used censoring schemes. Censoring schemes are classified into several categories, including Type I, Type II, and type II progressive censoring. In Type I (II) censoring schemes, the experiment terminated at a predetermined time (number of units). While type-II progressively censored samples allow researchers to remove units during the experiment. So in this paper, we compare the behavior of different types of estimators considered under type II progressive censoring for previously determined elimination units in each stage of type II progressive censoring (fixed removal) compared to unknown number of elimination units (Discrete uniform and binomial removal). The [20] provided how to employ type II progressive censoring in your code. Many authors used type II progressive censoring in their contribution as [21–23].

This article explores the estimation of parameters for the q−GEVL distribution employing MLE and the bayesian estimation BE for a type-II progressively censored sample. Since the resulting equations of MLE are not easy to solve by classical techniques like Newton. We utilize the particle swarm optimization (PSO) Algorithm to handle the difficulty of MLE.

PSO is considered One of the most popular algorithms in optimization. which is defined as a computational technique for solving optimization problems. It is inspired by the social acts of birds swarming or fish swarming. It is a population-based stochastic optimization technique that simulations particle movements (possible solutions) in a search space to determine the best solution to a problem. Each particle in the swarm represents a possible solution, which advances through the search space by following the swarm’s current best solution. Particles eventually converge on the optimum answer based on their own and their neighbors’ experiences see, [24] for more details. Their are many advantages of PSO such as, effectiveness for Multi-Modal Problems, fast Convergence, generally robust, less sensitive to parameter and it does not require gradient information.

The aims of the proposed article can be given as following:

- parameters estimation of q−GEVL under Type II censoring schemes using both MLE and BE.
- introducing PSO optimization techniques for MLE.
- Trying to use the fitting to predict some of the future measurements.

The article is organized as follows: In Sect 2, we investigate the properties of the q−GEVL distribution. Sect 3, we outlines the three cases under investigation and investigated MLE of q−GEVL parameters based on type-II progressively censored samples for the three cases of removals. Moreover, approximate confidence intervals are utilize. In Sect 4, BE for both non-informative and informative priors using two different loss functions. In Sect 5, a simulation is purposed to as an application of the theoretical parts in this paper. Sect 6 contains the real data example. Finally, Sect 7 summarizes the article’s findings.

## 2 q−GEVL Distribution properties

This section discusses the effect of the parameter *q* on the behavior of q−GEVL the distribution, the probability density function, PDF , and the cumulative density function , CDF. Moreover, the distribution properties such as the quantile function, return level, reversed hazard rate, hazard rate, moments, skewness and kurtosis and the moment generating function.

### 2.1 q−GEVL PDF and CDF

**Fig 1:**
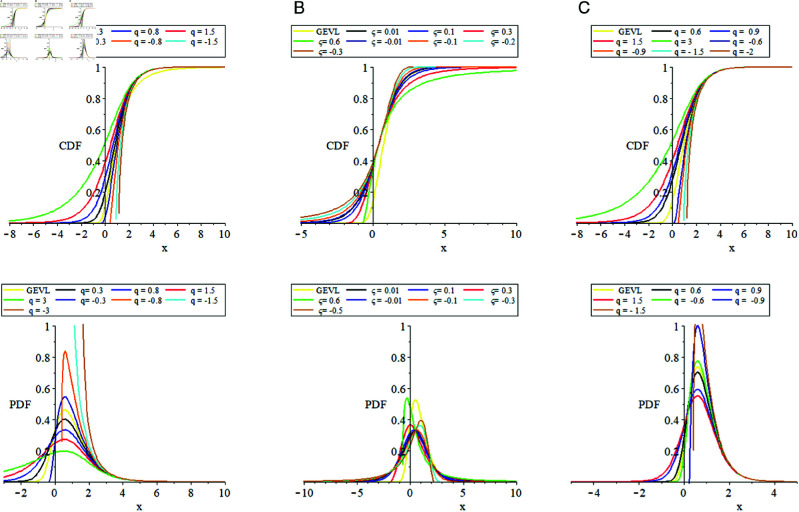
The plots of q−GEVL distribution CDF and PDF. (a) (μ=0.6, σ=0.8, ξ=0.02), (b) (*q* = 1.3, μ=0.5, σ=0.7), (c) (μ=0.6, σ=0.9) and ξ→0.

Since, PDF and CDF of the q−GEVL according to [13] are given by

$$g_{q}(x;q,𝒮)=\left\{ \begin{array}{cc} \frac{{\left[1+\xi (\frac{x-\mu }{\sigma })\right]}^{-\frac{1}{\xi }-1}}{\sigma {\left[1+q{\left(\xi (\frac{x-\mu }{\sigma })+1\right)}^{-\frac{1}{\xi }}\right]}^{\frac{1}{q}+1}}, & \xi \neq 0,\\ \frac{e^{-(\frac{x-\mu }{\sigma })}}{\sigma {\left[1+qe^{-(\frac{x-\mu }{\sigma })}\right]}^{\frac{1}{q}+1}}, & \xi \to 0, \end{array}\right.$$ (1)

and

$$G_{q}(x;q,𝒮)=\left\{ \begin{array}{cc} {\left[1+q{\left(\xi (\frac{x-\mu }{\sigma })+1\right)}^{-\frac{1}{\xi }}\right]}^{-\frac{1}{q}}, & \xi \neq 0,\\ {\left[1+qe^{-(\frac{x-\mu }{\sigma })}\right]}^{-\frac{1}{q}}, & \xi \to 0, \end{array}\right.$$ (2)

where,

$$𝒮=\left\{ \begin{array}{cc} (\mu ,\sigma ,\xi ), & \text{if}\;\xi \neq 0\\ (\mu ,\sigma ), & \text{if}\;\xi \to 0. \end{array}\right.$$ (3)

and

$$x\in \left\{ \begin{array}{cc} \left(\frac{\mu }{\sigma }-\frac{1}{\xi \sigma },\infty \right), & q>0,\;\xi >0,\\ \left(-\infty ,\frac{\mu }{\sigma }-\frac{1}{\xi \sigma }\right), & q>0,\;\xi <0,\\ \left(\frac{{\left(-q\right)}^{\xi }-1}{\xi \sigma }+\frac{\mu }{\sigma },\infty \right), & q<0,\;\xi >0,\\ \left(\frac{{\left(-q\right)}^{\xi }-1}{\xi \sigma }+\frac{\mu }{\sigma },\frac{\mu }{\sigma }-\frac{1}{\xi \sigma }\right), & q<0,\;\xi <0,\\ \left(-\infty ,\infty \right), & \xi \to 0,\;q>0,\\ \left(\frac{\mu +\ln(-q)}{\sigma },\infty \right), & \xi \to 0,\;q<0. \end{array}\right.$$ (4)

to investigate the impact of the parameters qand𝒮 on the shapes of the distribution both CDF and PDF are plotted in Fig 1 using different sets of parameters.

Fig 1 shows,

- The parameter combinations (q,μ,σ,ξ) strongly influence the shapes of both the CDF and PDF.
- The CDF graphs reflect distributions of probability accumulates continuously as *x* increases. The changes in these curves indicate how probability dependent on parameter values.
- The PDF variations represent deviations in the distribution’s skewness and kurtosis, with some plots showing sharp peaks and heavy tails and others being more spread out.
- The parameter ξ controls the tail behavior, while *q* controls the overall height and dispersion of the curves.

### 2.2 Moments

Both ${\text{k}}^{\text{th}}$moments (μk′) and Moment Generating Function (MGF) are essential concepts in statistics and probability theory that characterize the probability distributions. Moreover, they are important features in theoretical and practical situations since it aids in summarizing and evaluating data distribution. The μk′ indicate many characteristics of a random variable, including as its central tendency, shape, variability, and tail behavior. While the MGF can be used to derive all moments of a random variable (such as the mean, variance, skewness, and higher-order moments).

Let *X* be any random variable (RV) follows q−GEVL then the ${\text{k}}^{\text{th}}$ moments and MGF are


μk′=E[Xk]=∫−∞∞xkgq(x;q,𝒮)dxE[exp(tx)]=∫−∞∞exp(tx)gq(x;q,𝒮)dx

Then

$$E[X^{k}]=\left\{ \begin{array}{cc} \int_{x}\frac{x^{k}{\left(1+\xi (\frac{x-\mu }{\sigma })\right)}^{-\frac{1}{\xi }-1}}{\sigma {\left[1+q{\left(\xi (\frac{x-\mu }{\sigma })+1\right)}^{-\frac{1}{\xi }}\right]}^{\frac{1}{q}+1}}dx, & \xi \neq 0,\;\\ \int_{x}\frac{x^{k}e^{\left(\frac{x-\mu }{\sigma }\right)}}{\sigma {\left[1+qe^{\left(\frac{x-\mu }{\sigma }\right)}\right]}^{\frac{1}{q}+1}}dx, & \xi \to 0, \end{array}\right.$$ (5)

$$E[\exp(tx)]=\left\{ \begin{array}{cc} \int_{x}\frac{\exp(tx){\left(1+\xi (\frac{x-\mu }{\sigma })\right)}^{-\frac{1}{\xi }-1}}{\sigma {\left[1+q{\left(\xi (\frac{x-\mu }{\sigma })+1\right)}^{-\frac{1}{\xi }}\right]}^{\frac{1}{q}+1}}dx, & \xi \neq 0,\;\\ \int_{x}\frac{e^{\left(tx+\frac{x-\mu }{\sigma }\right)}}{\sigma {\left[1+qe^{\left(\frac{x-\mu }{\sigma }\right)}\right]}^{\frac{1}{q}+1}}dx, & \xi \to 0, \end{array}\right.$$ (6)

where the support of *x* given in Eq 4.

It’s clear from Eqs 5–(6) that these equations cannot be reduced to a closed form. So, the numerical software is used to evaluate these integrations using specified parameter values;

see Table 1.

**Table 1:** The statistical properties of q−𝒢ℰ𝒱ℒ and 𝒢ℰ𝒱ℒ at and (q=0.5,μ=0.5,σ=0.2) respectively.

			(μ1′,μ2′,μk′)	*Var*(*x*)	$\left({𝒬}_{0.25},{𝒬}_{0.5},{𝒬}_{0.75}\right)$	(${𝒵}_{10}$,${𝒵}_{20}$,${𝒵}_{100}$)	SK	KR
GEVL	ξ≠0	(μ=0.5,σ=0.2,ξ=0.01)	(0.9902,0.9595,1.1967)	0.9902	(0.4357,0.57466,0.7653)	(1.0047,1.1916,1.6681)	–0.76768	0.6925
-9	ξ→0	(μ=0.5,σ=0.2)	(0.1231,0.0889,0.0748)	0.0616	(0.4346,0.5733,0.7491)	0.95007,1.0940,1.4200)	3.0811	11.3545
q−GEVL	ξ≠0	(q=0.5,μ=0.5,σ=0.2,ξ=0.01)	(–1.77*10^−14^,5.75811*10^−14^,–1.298076*10^−13^)	5.75811*10^−14^	(0.3618,0.5376,0.7360)	(0.9498,1.1003,1.4409)	2.568019*10^ + 19^	1.082264*10^ + 26^
-9	ξ→0	(q=0.5,μ=0.5,σ=0.2)	(0.6104,0.4404,0.3709)	0.0678	(0.4277,0.5698,0.7477)	(0.9495,1.0937,1.419)	1.0888	5.2592

### 2.3 Quantile function, skewness, and kurtosis

The quantile function (${𝒬}_{P}$) is a fundamental concept in statistics that explains the connection between probabilities and the corresponding values for a random variable. It is essentially the inverse of CDF and is used to determine the minimum value at which a particular proportion of the data falls. According to [13] the quantile function is


$${𝒬}_{P}=\left\{ \begin{array}{cc} \mu +\frac{\sigma }{\xi }\left[{\left(\frac{p^{-q}-1}{q}\right)}^{-\xi }-1\right], & \xi \neq 0,\\ \mu -\sigma \ln\left(\frac{p^{-q}-1}{q}\right), & \xi \to 0. \end{array}\right.$$

Since the Skewness S𝒦 details the asymmetry or lack of symmetry in the data. While kurtosis 𝒦R, helps measure the presence of outliers by assessing the heaviness of tails and the sharpness of the peak. The commonly used formals of SK and KR can be obtained from the distribution’s moments as in the formula 2 in Eqs 7 and (8), specifically the third moment (for skewness) and the fourth moment (for kurtosis), or by depending on the quantile function as given by the formula of Bowley’s Skewness and Moors’ kurtosis as in the formula 1 in Eqs 7 and (8). Where Bowley’s Skewness is a quantile-based skewness measurement that quantifies a distribution’s skew by employing Quartiles. While Moors’ kurtosis is a quantile-based measure of kurtosis, which provides a strong alternative to classic moment-based kurtosis. Using Octiles minimizes sensitivity to extreme outliers and offers a more consistent approximation of a distribution’s tails. Then S𝒦 and 𝒦R are given as

$$S𝒦=\left\{ \begin{array}{cc} \frac{{𝒬}_{\frac{3}{4}}+{𝒬}_{\frac{1}{4}}-2{𝒬}_{\frac{1}{2}}}{{𝒬}_{\frac{3}{4}}-{𝒬}_{\frac{1}{4}}} & \text{Formula 1}\\ \frac{E\left[(X-\mu {)}^{3}\right]}{[var(x){]}^{\frac{3}{2}}} & \text{Formula 2} \end{array}\right.$$ (7)

$$𝒦R=\left\{ \begin{array}{cc} \frac{{𝒬}_{\frac{7}{8}}-{𝒬}_{\frac{1}{8}}+{𝒬}_{\frac{7}{8}}-{𝒬}_{\frac{5}{8}}}{{𝒬}_{\frac{6}{8}}-{𝒬}_{\frac{2}{8}}}, & \text{Formula 1}\\ \frac{E\left[(X-\mu {)}^{4}\right]}{[var(x){]}^{2}} & \text{Formula 2} \end{array}\right.$$ (8)

Then S𝒦 and 𝒦R, could be easily obtained as

1. For ξ≠0
  - $$S𝒦=\left\{ \begin{array}{cc} \frac{((\frac{3}{4}{)}^{-q}-1{)}^{-\xi }+((\frac{1}{4}{)}^{-q}-1{)}^{-\xi }-2((\frac{1}{4}{)}^{-q}-1{)}^{-\xi }}{((\frac{3}{4}{)}^{-q}-1{)}^{-\xi }-((\frac{1}{4}{)}^{-q}-1{)}^{-\xi }} & \text{Formula 1}\\ \int_{-\infty }^{\infty }\frac{(X-\mu {)}^{3}{\left(1+\xi (\frac{x-\mu }{\sigma })\right)}^{-\frac{1}{\xi }-1}}{[var(x){]}^{\frac{3}{2}}\sigma {\left[1+q{\left(\xi (\frac{x-\mu }{\sigma })+1\right)}^{-\frac{1}{\xi }}\right]}^{\frac{1}{q}+1}}dx, & \text{Formula 2} \end{array}\right.$$
  - $$𝒦R=\left\{ \begin{array}{cc} \frac{((\frac{3}{8}{)}^{-q}-1{)}^{-\xi }-((\frac{1}{8}{)}^{-q}-1{)}^{-\xi }+({(\frac{7}{8}{)}^{-q}-1}^{-\xi })-((\frac{5}{8}{)}^{-q}-1{)}^{-\xi }}{((\frac{6}{8}{)}^{-q}-1{)}^{-\xi }-((\frac{2}{8}{)}^{-q}-1{)}^{-\xi }}, & \text{Formula 1}\\ \int_{-\infty }^{\infty }\frac{(X-\mu {)}^{4}{\left(1+\xi (\frac{x-\mu }{\sigma })\right)}^{-\frac{1}{\xi }-1}}{[Var(x){]}^{2}\sigma {\left[1+q{\left(\xi (\frac{x-\mu }{\sigma })+1\right)}^{-\frac{1}{\xi }}\right]}^{\frac{1}{q}+1}}dx, & \text{Formula 2} \end{array}\right.$$
2. For ξ→0
  - $$S𝒦=\left\{ \begin{array}{cc} \frac{\log(\frac{(\frac{3}{4}{)}^{-q}-1}{q})+\log(\frac{(\frac{1}{4}{)}^{-q}-1}{q})-2\log(\frac{(\frac{1}{4}{)}^{-q}-1}{q})}{\log(\frac{(\frac{3}{4}{)}^{-q}-1}{q})-\log(\frac{(\frac{1}{4}{)}^{-q}-1}{q})} & \text{Formula 1}\\ \int_{-\infty }^{\infty }\frac{(X-\mu {)}^{3}e^{\left(\frac{x-\mu }{\sigma }\right)}}{[var(x){]}^{\frac{3}{2}}\sigma {\left[1+qe^{\left(\frac{x-\mu }{\sigma }\right)}\right]}^{\frac{1}{q}+1}}, & \text{Formula 2} \end{array}\right.$$
  - $$𝒦R=\left\{ \begin{array}{cc} \frac{\log(\frac{(\frac{3}{8}{)}^{-q}-1}{q})-\log(\frac{(\frac{1}{8}{)}^{-q}-1}{q})+\log(\frac{(\frac{7}{8}{)}^{-q}-1}{q})-\log(\frac{(\frac{5}{8}{)}^{-q}-1}{q})}{\log(\frac{(\frac{6}{8}{)}^{-q}-1}{q})-\log(\frac{(\frac{2}{8}{)}^{-q}-1}{q})}, & \text{Formula 1}\\ \int_{-\infty }^{\infty }\frac{(X-\mu {)}^{4}e^{\left(\frac{x-\mu }{\sigma }\right)}}{[var(x){]}^{2}\sigma {\left[1+qe^{\left(\frac{x-\mu }{\sigma }\right)}\right]}^{\frac{1}{q}+1}},, & \text{Formula 2} \end{array}\right.$$

**Fig 2:**
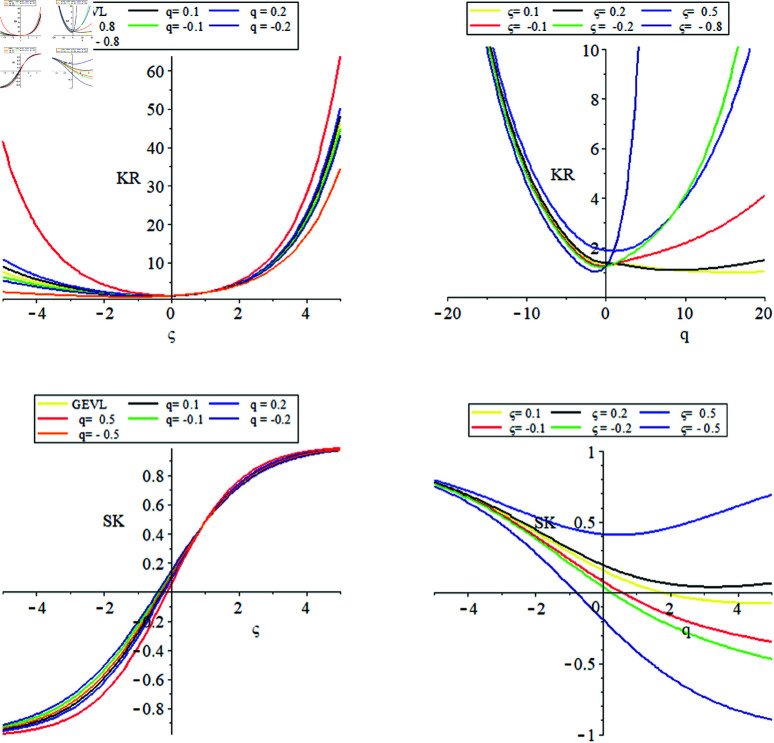
The plots of q−GEVL for the SK and KR at (μ=0.5,σ=1) for different values of q and ξ.

Fig 2 are purposed for a better understanding of the effect of parameters *q* and ξ on the behavior of the S𝒦 and 𝒦R

From Fig 2:

1. S𝒦 and 𝒦R change non-linearly with respect to the parameters ξ and *q*, where the scaling parameter affecting the distribution properties. Since a larger scale parameter results in a wider spread and contrary a smaller scale parameter results in a narrower distribution.
2. The 𝒦R increaseing sharply for larger values of ξ, particularly for positive ξ, indicating fatter tails in the distribution.
3. The tail behaviour of S𝒦 is effected by the values of ξ, where positive ξ leads to positive S𝒦 with longer right tail where as negative ξ leads to negative S𝒦 and longer left tail.

### 2.4 The return level

The return level ${𝒵}_{t}$is an important concept in the study of extreme value theory (EVT), particularly in applications such as hydrology, climate science, finance, and engineering, where we want to evaluate the size of an extreme event that is predicted to occur over a given time period. Let *T* be the return period, then mathematically, the return level ${𝒵}_{t}$ is given by $P(X>{𝒵}_{t})=\frac{1}{T}$. Then $1-G_{q}({𝒵}_{t};q,𝒮)=\frac{1}{T}$. Then


$$\frac{1}{T}=\left\{ \begin{array}{cc} 1-{\left[1+q{\left(\xi (\frac{{𝒵}_{t}-\mu }{\sigma })+1\right)}^{-\frac{1}{\xi }}\right]}^{-\frac{1}{q}}, & \xi \neq 0,\\ 1-{\left[1+qe^{-(\frac{{𝒵}_{t}-\mu }{\sigma })}\right]}^{-\frac{1}{q}}, & \xi \to 0, \end{array}\right.$$

After some calculation, it is easy to conduct,


$${𝒵}_{t}=\left\{ \begin{array}{cc} \mu +\frac{\sigma }{\xi }[[\frac{[1-(\frac{1}{T}{)}^{-q}]-1}{q}{]}^{-\xi }-1], & \xi \neq 0\\ \mu -\sigma \log(\frac{[1-(\frac{1}{T}{)}^{-q}]-1}{q}), & \xi \neq 0 \end{array}\right.$$

Table 1 demonstrate the values of ${𝒵}_{t}$ for some parameter values.

From Table 1

- For *GEVL*, the median and quartiles are higher when ξ≠0 compared to ξ→0.
- For q−GEVL, the median and quartiles are significantly lower when ξ≠0, indicating a shift in central tendency.
- The variance remains relatively small for all cases, with q−GEVL compared to *GEVL*.
- For *GEVL*, *Z*_*t*_ values remain relatively consistent across different values of ξ. While for q−GEVL, the return levels are slightly higher compared to *GEVL*, suggesting heavier tails and greater tail dependence.
- *GEVL* exhibits negative skewness, indicating a left-skewed distribution, with a kurtosis, suggesting a platykurtic nature. While q−GEVL exhibits larger absolute skewness, implying a stronger right-skew. Then q−GEVL distribution modifies the tail behavior of *GEVL*, making it more sensitive to extreme values and left-skewed distributions
- In the case of the return levels (*Z*_*t*_) under these parameters, there is no noticeable effect between the two distributions.
- The result conducted from Table 1 is compatible with the result of real data in Sect 6 demonstrated in Table 9.

The result in Table 1, q−GEVL is particularly useful for scenarios where modeling extreme lower-end behavior as financial risk and environmental extremes are critical.

### 2.5 The hazard rate and reversed hazard rate

The hazard rate (HR) and reversed hazard rate (RHR) are two essential concepts in reliability theory and survival analysis. They describe several features of a mechanism’s or individual’s long-term failure or survival. Since the HR and RHR for q−GEVL distribution are ${HR}_{q}(x;q,𝒮)=\frac{g_{q}(x;q,𝒮)}{1-G_{q}(x;q,𝒮)}$ and ${RHR}_{q}(x;q,𝒮)=\frac{g_{q}(x;q,𝒮)}{G_{q}(x;q,𝒮)}$ respectively. By using Eqs 1–(2), it’s easy to get


$${HR}_{q}(x;q,𝒮)=\left\{ \begin{array}{cc} \frac{{\left(1+\xi (\frac{x-\mu }{\sigma })\right)}^{-\frac{1}{\xi }-1}{\left[1+q{\left(\xi (\frac{x-\mu }{\sigma })+1\right)}^{-\frac{1}{\xi }}\right]}^{\frac{-1}{q}-1}}{\sigma \left[1-{\left[1+q{\left(\xi (\frac{x-\mu }{\sigma })+1\right)}^{-\frac{1}{\xi }}\right]}^{-\frac{1}{q}}\right]}, & \xi \neq 0\\ \frac{e^{-(\frac{x-\mu }{\sigma })}{\left[1+qe^{\left(\frac{x-\mu }{\sigma }\right)}\right]}^{-\frac{1}{q}-1}}{\sigma \left[1-{\left[1+qe^{-(\frac{x-\mu }{\sigma })}\right]}^{-\frac{1}{q}}\right]}, & \xi \to 0 \end{array}\right.$$

and


$${RHR}_{q}(x;q,𝒮)=\left\{ \begin{array}{cc} \frac{{\left(1+\xi (\frac{x-\mu }{\sigma })\right)}^{-\frac{1}{\xi }-1}}{\sigma \left[1+q{\left(\xi (\frac{x-\mu }{\sigma })+1\right)}^{-\frac{1}{\xi }}\right]}, & \xi \neq 0\\ \frac{e^{-(\frac{x-\mu }{\sigma })}}{\sigma \left[1+qe^{-(\frac{x-\mu }{\sigma })}\right]}, & \xi \to 0 \end{array}\right.$$

**Fig 3:**
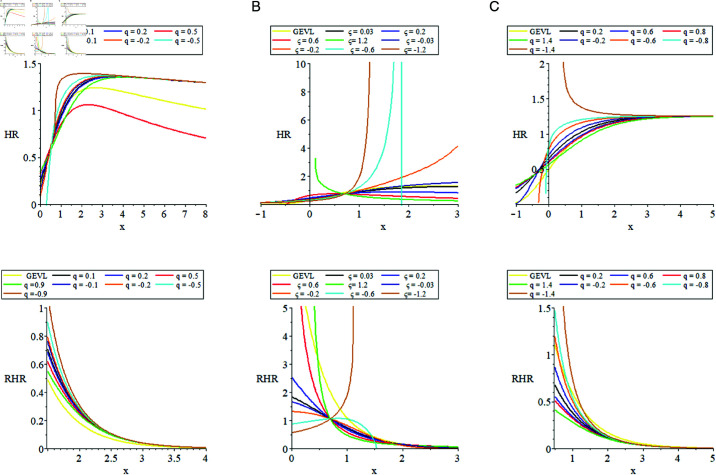
The plots of q−𝒢ℰ𝒱ℒ distribution ℋℛ and ℛℋℛ. (a) (μ=0.1, σ=0.5, ξ=0.01), (b) (*q* = 0.9, μ=0.7, σ=0.7), (c) (μ=0.1, σ=0.8) and ξ→0.

In Fig 3 we display some plots of HR and RHR for some parameter values.

From Fig 3, it’s clear that the hazard rate can rise or reduce periodically, depending on the parameter values, reflecting the various risks of systems or occurrences. In most circumstances, the RHR decreases, with small values of ξ. While HR shows increasing for the small value of ξ.

## 3 MLE of q−GEVL under progressive Type-II Censoring with different types removals

In this section, the MLE of q−GEVL parameters using PSO is displayed for both point and interval estimation under three cases of type II progressive censoring (Fixed, Discrete uniform, Binomial) random removal. The interval estimation of q−GEVL parameters is conducted using the Fisher information matrix.

### 3.1 Types removals of Type II progressive censoring

Let $\overset{ˇ}{R}=(R_{1},R_{2},\ldots ,R_{n})$ be vector of type II progressive censoring scheme. Then joint PDF of $\overset{ˇ}{R}$ is

$$P(\overset{ˇ}{R})=\left\{ \begin{array}{cc} \text{constant} & \text{ For fixed removal}\\ {}[3pt]\frac{1}{\left(N-n-1\right)}\prod_{i=1}^{n-1}\frac{1}{N-n-(\sum_{k=1}^{i-1}r)+1} & \text{For discrete uniform}\\ {}[6pt]\frac{(N-n)!P^{\sum_{i=1}^{n-1}r}(1-P{)}^{\left[(N-n)(n-1)-\sum_{i=1}^{n-1}(n-i)r\right]}}{\left(N-n-\sum_{i=1}^{n-1}r)!(\prod_{i=1}^{n-1}r!\right)} & \text{For Binomial} \end{array}\right.$$ (9)

Where, 0<*r*_1_<*N*−*n*, $0<r_{i}<N-n-\sum_{k=1}^{i-1}r_{k}$, for i=2,3,…,n−1, $R_{n}=N-n-\sum_{k=1}^{n-1}R_{k}$. For more details on how to obtain Eq 9 see, [25].

### 3.2 MLE of q=−GEVL distribution parameters

Let *X* be a random variable following q−GEVL distribution. Then the joint likelihood function based on Type II progressive censored data $\overset{ˇ}{R}$ is given by $L^{*}=P(\overset{ˇ}{R})L$. Then


$$L^{*}\propto \left\{ \begin{array}{cc} L(q;S), & \text{ For fixed removal}\\ L(q;S), & \text{For discrete uniform}\\ \frac{L(q;S)P^{\sum_{i=1}^{n-1}r_{i}}}{(1-P{)}^{-(n-1)(N-n)+\sum_{i=1}^{n-1}(n-i)r_{i}}}, & \text{For binomial} \end{array}\right.$$

where

$$L(q;S)=C\prod_{i=1}^{n}g(x_{i};q,𝒮)[1-G(x_{i};q,𝒮){]}^{r_{i}},$$ (10)

where, *S* is defined at Eq 3 and $C=N\prod_{j=2}^{n-1}\left(N-\sum_{i=1}^{j-1}r_{i}-i+1\right)$. By using Eqs (1–(2) in Eq (10), we obtain

$$L(q;S)\propto \left\{ \begin{array}{cc} \prod_{i=1}^{n}\frac{{\left(1+\xi (\frac{x_{i}-\mu }{\sigma })\right)}^{-\frac{1}{\xi }-1}\times {\left[1+q{\left(\xi (\frac{x_{i}-\mu }{\sigma })+1\right)}^{-\frac{1}{\xi }}\right]}^{-\frac{1}{q}-1}}{\sigma {\left[1-{\left[1+q{\left(\xi (\frac{x_{i}-\mu }{\sigma })+1\right)}^{-\frac{1}{\xi }}\right]}^{-\frac{1}{q}}\right]}^{-r_{i}}}, & \xi \neq 0\\ \prod_{i=1}^{n}\frac{e^{-(\frac{x_{i}-\mu }{\sigma })}{\left[1+qe^{-(\frac{x_{i}-\mu }{\sigma })}\right]}^{-\frac{1}{q}-1}}{\sigma {\left[1-{\left[1+qe^{-(\frac{x_{i}-\mu }{\sigma })}\right]}^{-\frac{1}{q}}\right]}^{-r_{i}}}, & \xi \to 0 \end{array}\right.$$ (11)

then the MLE of parameters of q−GEVL are,

1. ξ≠0
  i.
    $$\frac{\partial L(q;S)}{\partial q}=\sum_{i=1}^{n}\frac{\ln(1+qf(S))}{q^{3}}-\sum_{i=1}^{n}\frac{\left[\frac{\ln[1+qf(S)]}{q^{2}}-\frac{f(s)}{q[1+qf(S)]}\right]}{[\frac{q+1}{q}-\frac{r_{i}[1-[1+qf(S){]}^{\frac{-1}{q}}]}{[1+qf(S){]}^{\frac{-1}{q}}}{]}^{-1}}$$ (12)
  ii.
    $$\frac{\partial L(q;S)}{\partial \mu }=\sum_{i=1}^{n}\frac{(q+1)f(S{)}^{1+\xi }}{q[1+qf(S)]}+\sum_{i=1}^{n}(\xi +1)f(S{)}^{\xi }\;-\sum_{i=1}^{n}\frac{r_{i}f(S{)}^{1+\xi }[1+qf(S){]}^{-1-\frac{1}{q}}}{{\left[1-[1+qf(S)]\right]}^{\frac{-1}{q}}}$$ (13)
  iii.
    $$\frac{\partial L(q;S)}{\partial \sigma }=-n\sigma +\sum_{i=1}^{n}\frac{(q+1)(x_{i}-\mu )f(S{)}^{1+\xi }}{q[1+qf(S)]}\;+\sum_{i=1}^{n}\frac{\left(\xi +1)(x_{i}-\mu \right)}{f(S{)}^{-\xi }}-\sum_{i=1}^{n}\frac{r_{i}(x_{i}-\mu )[1+qf(S){]}^{-1-\frac{1}{q}}}{f(S{)}^{-(1+\xi )}{\left[1-[1+qf(S)]\right]}^{\frac{-1}{q}}}$$ (14)
  iv.
    $$\frac{\partial L(q;S)}{\partial \xi }=-\sum_{i=1}^{n}\frac{(q+1)f(S)\left[\ln(f(S))+\frac{\left(x_{i}-\mu \right)}{\sigma f(S{)}^{-\xi }}\right]}{q[1+qf(S)]}-\sum_{i=1}^{n}\frac{\left(\xi +1)(x_{i}-\mu \right)}{\sigma f(S{)}^{-\xi }}\;-\sum_{i=1}^{n}\frac{r_{i}f(S)\left[\ln(f(S))-\frac{\left(x_{i}-\mu \right)}{\sigma f(S{)}^{-\xi }}\right]}{\left[1-[1+qf(S){]}^{\frac{-1}{q}}\right][1+qf(S){]}^{1+\frac{1}{q}}}$$ (15)
2. ξ→0
  i.
    $$\frac{\partial L(q;S)}{\partial q}=\sum_{i=1}^{n}\frac{\ln(1+qf(S))}{q^{3}}-\sum_{i=1}^{n}\frac{\left[\frac{\ln[1+qf(S)]}{q^{2}}-\frac{f(s)}{q[1+qf(S)]}\right]}{[\frac{q+1}{q}-\frac{r_{i}[1-[1+qf(S){]}^{\frac{-1}{q}}]}{[1+qf(S){]}^{\frac{-1}{q}}}{]}^{-1}}$$ (16)
  ii.
    $$\frac{\partial L(q;S)}{\partial \mu }=\sum_{i=1}^{n}\frac{\left(q+1)f(S\right)}{q[1+qf(S)]}+n-\sum_{i=1}^{n}\frac{r_{i}f(S)[1+qf(S){]}^{-1-\frac{1}{q}}}{{\left[1-[1+qf(S)]\right]}^{\frac{-1}{q}}}$$ (17)
  iii.
    $$\frac{\partial L(q;S)}{\partial \sigma }=-n+\sum_{i=1}^{n}\frac{\left(q+1)(x_{i}-\mu )f(S\right)}{q[1+qf(S)]}+\sum_{i=1}^{n}\frac{\left(x_{i}-\mu \right)}{\sigma }\;-\sum_{i=1}^{n}\frac{r_{i}(x_{i}-\mu )[1+qf(S){]}^{-1-\frac{1}{q}}}{f(S){\left[1-[1+qf(S)]\right]}^{\frac{-1}{q}}}$$ (18)

where


$$f(S)=\left\{ \begin{array}{cc} {\left(\xi (\frac{x_{i}-\mu }{\sigma })+1\right)}^{-\frac{1}{\xi }}, & \xi \neq 0\\ e^{-(\frac{x_{i}-\mu }{\sigma })}, & \xi \to 0 \end{array}\right.$$

Since the Eqs 12–(15) and Eqs 16)–(18) are very difficult and challenging to solve analytically and numerically by the well known traditional methods, the PSO is suggested to handeled this probem. Statisticians and researchers can easily incorporate it into their code by following the steps outlined in ([26]).

For the implementation of PSO follow these steps:

1. Define the log-likelihood function, log(L(q,S)) which called Fitness function.
2. Specify the search interval for each parameter.
3. Execute the optimization using the (psoptim) function in R.

### 3.3 Fisher Information Matrix (FIM)

The Fisher Information Matrix (FIM) is a key principle in statistical estimation and information theory that helps in the evaluation of the parameters’ confidence interval. FIM is equal to the matrix of the negative values of the second partial derivatives of the logarithm of the joint likelihood function given at Eq 10. Then,


$$ℐ(S)=-ℰ\left[\frac{\partial^{2}\log L(\hat{q};\hat{S})}{\partial {\hat{\theta }}^{2}}\right]$$

where, *S* is given at Eq 3, $\hat{\theta }\in \{\hat{S},\hat{q}\}$ and $\hat{\theta }$ is the MLE of θ. Since the variance-covariance matrix of the parameters is the inverse of FIM.

Hance the parameter θ is normally distributied with mean $\hat{\theta }$ and variance $Var(\hat{\theta })\approx {ℐ}^{\left(-1\right)}(S)$. Then the confidence interval of the parameter θ is given by $\hat{\theta }\pm {𝒵}_{\frac{\alpha }{2}}\sqrt{Var(\hat{\theta })}$.

## 4 Bayesian estimation BE

In this section we considered the parameters Bayesian estimation (BE) of q−GEVL for both informative and non-informative cases under the three cases of Type II progressive censoring discussed above using two different loss functions square error loss function (sq) and linex loss function (lx).

**Informative BE**

For this case suppose that, all the parameters in *S* are following exponential distribution with different hyperparameters (*b*1–*b*4). While, the prior PDF of *P* follows beta distribution with hyperparameters *c*,*d*. Then the joint prior PDF could be written as


$$\Phi (q,S)\propto \left\{ \begin{array}{cc} e^{-(b_{1}q+b_{2}\mu +b_{3}\sigma +b_{4}\xi )} & \text{For fixed and discrete uniform}\\ p^{1-c}(1-p{)}^{1-d}e^{-(b_{1}q+b_{2}\mu +b_{3}\sigma +b_{4}\xi )} & \text{For binomial} \end{array}\right.$$

the method of selecting the PDF prior is purposed in [25] and used at [27]. Then the joint posterior PDF is


$$\Phi^{*}(q,S)\propto \left\{ \begin{array}{cc} e^{-(b_{1}q+b_{2}\mu +b_{3}\sigma +b_{4}\xi )}L(q,S) & \text{For fixed and discrete uniform}\\ \frac{p^{1-C+\sum_{i=1}^{n-1}r_{i}}(1-p{)}^{1-d-(n-1)(N-n)+\sum_{i=1}^{n-1}(n-i)r_{i}}L(q,S)}{e^{\left(b_{1}q+b_{2}\mu +b_{3}\sigma +b_{4}\xi \right)}} & \text{For binomial} \end{array}\right.$$

where, *L*(*q*,*S*) given at Eq 10.

**Non-informative BE**

For this case suppose that all the parameters follow a uniform distribution with interval [0,1]. Then the joint prior PDF is equal 1. Then the joint posterior PDF is proportional with *L* given at Eq 10. Let θ∈{q,S}, then for both cases of the BE using LINEX (*lx*) and square error loss function (*sq*) is given by

$$\tilde{\theta }=\left\{ \begin{array}{cc} E(\theta ), & \text{For}\;sq\\ \frac{-1}{\beta }\log\;E(\exp(-\beta \theta )), & \text{For}\;lx \end{array}\right.$$ (19)

where the expectation in Eq 19 is taking with the respect of the joint posterior PDF. For more details on loss functions given at Eq 19, see [25], which is mentioned in detail. Since this expectation in Eq 19 cannot be reduced analytically, Lindley’s approximation is used; see [28].

## 5 Simulation

In this section, a Monte Carlo simulation is used to evaluate the productivity of numerous parameter estimators that have been mentioned earlier assuming the real values of parameters are (0.1,0.5,0.5,0.2). To test the sensitivity of claimed estimator techniques to sample size, datasets of 1000 samples are produced using different sample sizes 50,100,200,500and1000. Further, Lindley’s approximation technique is used to compute BE for both *sq* and *lx* loss functions for three different values of the parameter β, namely {-1, 1, 0.5} with informative (*inf*)and non-informative (*non*) priors. The estimators are thoroughly tested utilizing *Bias* and mean squared error (*MSE*) analysis. Additionally, a 95% confidence interval for the parameters is determined. Tables 2, 3, 4, and 5 display the *Bias* and *MSE* of multiple estimators for the parameter (q=0.1,μ=0.5,σ=0.5,ξ=0.2) under three distinct scenarios of removals of type II progressive censoring. Moreover, the results of the confidence interval for these parameters are proposed in terms of upper bound( UB ), lower bound( LB ) and length of the interval bound ( IL ).

**Table 2:** The results for the fixed removal.

			*Bais*					*MSE*				
			*n* = 50	*n* = 100	*n* = 200	*n* = 500	*n* = 1000	*n* = 50	*n* = 100	*n* = 200	*n* = 500	*n* = 1000
*q*	*MLE*		0.02390	0.02420	0.02522	0.02799	0.02714	0.00146	0.00147	0.00151	0.00162	0.00159
	*non*	*sq*	0.06030	0.04631	0.03990	0.03358	0.03059	0.02967	0.00627	0.00267	0.00143	0.00139
		*lx*_(0.5)_	0.12945	0.08101	0.05961	0.04065	0.03469	0.18498	0.04012	0.01107	0.00224	0.00146
		*lx*_(−1)_	0.03730	0.03531	0.03407	0.03117	0.02941	0.02342	0.00561	0.00214	0.00119	0.00126
		*lx*_(1)_	0.03189	0.03109	0.02970	0.03018	0.02826	0.00370	0.00282	0.00223	0.00192	0.00174
	*inf*	*sq*	0.01634	–0.03204	–0.03139	0.00292	0.01520	0.26476	0.06494	0.01994	0.00107	0.00027
		*lx*_(0.5)_	0.13931	0.02216	–0.00325	0.01110	0.01959	0.52100	0.11476	0.03573	0.00256	0.00054
		*lx*_(−1)_	–0.17278	–0.08807	–0.05398	–0.00164	0.01349	0.42382	0.08748	0.02571	0.00128	0.00023
		*lx*_(1)_	0.04808	–0.03002	–0.03297	0.00072	0.01318	0.17589	0.02483	0.00888	0.00025	0.00029
μ	*MLE*		0.01772	0.01760	0.01574	0.01268	0.01292	0.00121	0.00120	0.00113	0.00101	0.00102
	*non*	*sq*	–0.01029	0.01042	0.01408	0.01232	0.01288	0.01247	0.00196	0.00136	0.00109	0.00105
		*lx*_(0.5)_	–0.03198	0.00245	0.01194	0.01178	0.01275	0.08494	0.00366	0.00176	0.00119	0.00109
		*lx*_(−1)_	–0.00819	0.01281	0.01518	0.01273	0.01308	0.01040	0.00227	0.00142	0.00111	0.00106
		*lx*_(1)_	0.01111	0.01488	0.01459	0.01227	0.01272	0.00085	0.00104	0.00106	0.00099	0.00101
	*inf*	*sq*	–0.01949	–0.00495	0.00004	0.00563	0.00952	0.05940	0.01095	0.00444	0.00175	0.00134
		*lx*_(0.5)_	–0.03318	–0.01103	–0.00165	0.00514	0.00940	0.19925	0.01369	0.00499	0.00188	0.00138
		*lx*_(−1)_	–0.03608	–0.00670	0.00029	0.00593	0.00969	0.06134	0.01651	0.00454	0.00178	0.00136
		*lx*_(1)_	0.01419	0.00177	0.00128	0.00567	0.00938	0.03690	0.00809	0.00393	0.00162	0.00129
σ	*MLE*		0.02402	0.02426	0.02540	0.02810	0.02726	0.00146	0.00147	0.00152	0.00162	0.00159
	*non*	*sq*	0.20027	0.10266	0.05702	0.03866	0.03225	0.11954	0.01182	0.00350	0.00196	0.00169
		*lx*_(0.5)_	0.21040	0.10298	0.05657	0.03843	0.03212	0.08224	0.01194	0.00346	0.00197	0.00169
		*lx*_(−1)_	0.19732	0.10197	0.05788	0.03912	0.03252	0.05661	0.01161	0.00357	0.00196	0.00168
		*lx*_(1)_	0.02198	0.02358	0.02522	0.02806	0.02725	0.00142	0.00147	0.00153	0.00163	0.00160
	*inf*	*sq*	0.23875	0.12981	0.08142	0.04842	0.03710	0.20745	0.02176	0.00882	0.00352	0.00240
		*lx*_(0.5)_	0.49458	0.21525	0.11351	0.05870	0.04187	0.57876	0.05261	0.01414	0.00425	0.00258
		*lx*_(−1)_	0.22495	0.12747	0.08141	0.04877	0.03734	0.10098	0.02047	0.00850	0.00348	0.00238
		*lx*_(1)_	0.08559	0.05402	0.05076	0.03797	0.03213	0.12043	0.01253	0.00732	0.00322	0.00232
ξ	*MLE*		0.02948	0.02780	0.02582	0.02299	0.02274	0.00168	0.00161	0.00153	0.00142	0.00141
	*non*	*sq*	–0.00120	0.02728	0.03002	0.02472	0.02391	0.09543	0.01002	0.00261	0.00178	0.00161
		*lx*_(0.5)_	–0.03208	0.02873	0.03381	0.02622	0.02496	0.09208	0.01473	0.00443	0.00222	0.00183
		*lx*_(−1)_	–0.00429	0.03336	0.03137	0.02525	0.02417	0.05379	0.00398	0.00269	0.00181	0.00163
		*lx*_(1)_	0.01923	0.02386	0.02429	0.02243	0.02248	0.00187	0.00138	0.00143	0.00139	0.00139
	*inf*	*sq*	–0.30044	–0.09323	–0.02859	0.00254	0.01308	0.65246	0.06599	0.00770	0.00211	0.00162
		*lx*_(0.5)_	–0.25376	–0.08077	–0.02285	0.00429	0.01419	0.38817	0.04542	0.01016	0.00265	0.00186
		*lx*_(−1)_	–0.34884	–0.10098	–0.03096	0.00261	0.01323	0.52442	0.04258	0.00833	0.00214	0.00163
		*lx*_(1)_	–0.20928	–0.08334	–0.03137	0.00065	0.01174	0.12817	0.01935	0.00528	0.00162	0.00138

**Table 3:** The results for the discrete uniform removal.

			Bais					MSE				
			*n* = 50	*n* = 100	*n* = 200	*n* = 500	*n* = 1000	*n* = 50	*n* = 100	*n* = 200	*n* = 500	*n* = 1000
*q*	MLE		0.00996	0.01198	0.01103	0.01299	0.01179	0.00328	0.00353	0.00337	0.00337	0.00347
	non	*sq*	0.05837	0.07219	0.06716	0.06423	0.05008	0.08659	0.02245	0.01580	0.01528	0.01132
		*lx*_(0.5)_	0.13083	0.16249	0.13922	0.12919	0.09763	0.17940	0.15705	0.06924	0.06602	0.04567
		*lx*_(−1)_	0.04036	0.05763	0.05856	0.05847	0.04654	0.03600	0.01940	0.01351	0.01276	0.00974
		*lx*_(1)_	0.01614	0.01741	0.01434	0.01433	0.01219	0.00468	0.00442	0.00380	0.00353	0.00354
	inf	*sq*	0.02685	0.00374	0.01790	0.04847	0.04512	0.33955	0.14970	0.05920	0.02181	0.01552
		*lx*_(0.5)_	0.17218	0.14451	0.11011	0.11697	0.09539	0.63122	0.38919	0.16455	0.08343	0.05876
		*lx*_(−1)_	–0.17238	–0.09576	–0.01601	0.03910	0.03954	0.54617	0.21206	0.05873	0.01760	0.01258
		*lx*_(1)_	0.04123	–0.01566	–0.02007	0.00139	0.00838	0.16341	0.07093	0.02897	0.00782	0.00518
μ	MLE		–0.00044	0.00211	0.00069	–0.00114	–0.00475	0.00407	0.00424	0.00397	0.00404	0.00412
	non	*sq*	0.00979	0.00386	0.00857	0.00881	0.00316	0.52800	0.00658	0.00473	0.00453	0.00450
		*lx*_(0.5)_	–0.03498	0.00557	0.01650	0.01911	0.01136	0.10427	0.01139	0.00683	0.00621	0.00576
		*lx*_(−1)_	–0.00968	0.00577	0.00943	0.00898	0.00320	0.02732	0.00683	0.00475	0.00453	0.00448
		*lx*_(1)_	–0.00655	–0.00046	–0.00046	–0.00157	–0.00497	0.00396	0.00413	0.00393	0.00403	0.00411
	inf	*sq*	–0.00287	–0.00955	–0.00080	0.00556	0.00217	0.36224	0.02066	0.00914	0.00547	0.00505
		*lx*_(0.5)_	–0.03018	–0.00484	0.00792	0.01593	0.01046	0.18724	0.02777	0.01180	0.00711	0.00644
		*lx*_(−1)_	–0.03245	–0.01398	–0.00126	0.00556	0.00209	0.06432	0.02847	0.00912	0.00544	0.00502
		*lx*_(1)_	–0.00264	–0.01051	–0.00869	–0.00462	–0.00589	0.05009	0.01517	0.00790	0.00500	0.00458
σ	MLE		–0.00201	0.00104	–0.00135	–0.00396	0.00129	0.00450	0.00449	0.00416	0.00410	0.00407
	non	*sq*	0.21867	0.06607	0.02362	–0.00022	0.00088	6.08077	0.01425	0.00492	0.00391	0.00398
		*lx*_(0.5)_	0.15224	0.06644	0.02299	–0.00056	0.00074	0.13668	0.01287	0.00477	0.00394	0.00400
		*lx*_(−1)_	0.14286	0.06109	0.02450	0.00052	0.00119	0.13374	0.04993	0.00621	0.00384	0.00393
		*lx*_(1)_	–0.00397	0.00052	–0.00152	–0.00400	0.00124	0.00454	0.00451	0.00419	0.00412	0.00407
	inf	*sq*	0.26044	0.09902	0.04270	0.00565	0.00323	4.88042	0.02734	0.00993	0.00494	0.00472
		*lx*_(0.5)_	0.41437	0.17178	0.06764	0.00888	0.00269	0.48226	0.05492	0.01340	0.00543	0.00509
		*lx*_(−1)_	0.17888	0.09223	0.04292	0.00646	0.00351	0.18949	0.04854	0.01079	0.00480	0.00466
		*lx*_(1)_	0.05868	0.03812	0.01917	0.00214	0.00370	0.05199	0.02041	0.01100	0.00579	0.00495
ξ	MLE		0.00488	0.00317	0.00203	–0.00011	0.00129	0.00380	0.00387	0.00397	0.00382	0.00403
	non	*sq*	0.02891	0.00969	0.01708	0.01386	0.01181	2.67338	0.01100	0.00533	0.00466	0.00458
		*lx*_(0.5)_	–0.04747	0.01931	0.03259	0.02842	0.02278	0.11821	0.02004	0.00915	0.00733	0.00639
		*lx*_(−1)_	–0.01845	0.01367	0.01787	0.01397	0.01182	0.05261	0.00693	0.00535	0.00464	0.00457
		*lx*_(1)_	–0.00454	–0.00023	0.00069	–0.00059	0.00103	0.00413	0.00376	0.00392	0.00380	0.00402
	inf	*sq*	–0.23423	–0.09917	–0.02958	–0.00106	0.00508	1.66572	0.06503	0.01222	0.00540	0.00485
		*lx*_(0.5)_	–0.24579	–0.07760	–0.01216	0.01365	0.01615	0.27707	0.05939	0.01668	0.00802	0.00676
		*lx*_(−1)_	–0.34834	–0.11542	–0.03246	–0.00130	0.00493	0.48010	0.07363	0.01285	0.00536	0.00482
		*lx*_(1)_	–0.20268	–0.09552	–0.04305	–0.01513	–0.00557	0.09643	0.02627	0.00950	0.00461	0.00422

**Table 4:** The results for the binomial removal.

			Bais					MSE				
			*n* = 50	*n* = 100	*n* = 200	*n* = 500	*n* = 1000	*n* = 50	*n* = 100	*n* = 200	*n* = 500	*n* = 1000
*q*	*MLE*		0.01201	0.01149	0.01209	0.01201	0.01496	0.00341	0.00351	0.00333	0.00353	0.00345
	*non*	*sq*	0.08480	0.06506	0.06643	0.06045	0.05193	0.04770	0.02217	0.01676	0.01430	0.01120
		*lx*_(0.5)_	0.19273	0.13509	0.00208	0.12189	0.09762	0.22481	0.08928	0.14727	0.06131	0.04324
		*lx*_(−1)_	0.06994	0.05435	0.13780	0.05491	0.04844	0.03304	0.01842	0.07422	0.01209	0.00967
		*lx*_(1)_	0.01222	0.01353	0.13207	0.01322	0.01549	0.00426	0.00406	0.35559	0.00372	0.00353
	*inf*	*sq*	0.16967	0.04912	0.05729	0.04841	0.03522	0.46807	0.25303	0.01491	0.02280	0.02551
		*lx*_(0.5)_	0.39854	0.21128	–0.08973	0.11523	0.08797	1.09560	0.58471	0.19164	0.08677	0.07634
		*lx*_(−1)_	–0.09943	–0.10233	0.01494	0.03848	0.02456	0.77773	0.35138	0.00380	0.01809	0.02032
		*lx*_(1)_	0.20090	0.07258	–0.00583	0.00364	0.00521	0.39309	0.18537	0.08824	0.00713	0.01454
μ	*MLE*		–0.00016	–0.00334	–0.00306	–0.00207	0.00031	0.00422	0.00390	0.00400	0.00400	0.00398
	*non*	*sq*	–0.02486	–0.00723	0.00132	0.00718	0.00801	0.02248	0.00624	0.00525	0.00463	0.00446
		*lx*_(0.5)_	–0.04086	–0.01122	–0.00987	0.01673	0.01599	0.07024	0.01124	0.01826	0.00638	0.00581
		*lx*_(−1)_	–0.02336	–0.00513	0.00549	0.00738	0.00804	0.02696	0.00640	0.00784	0.00463	0.00445
		*lx*_(1)_	–0.00662	–0.00608	–0.00289	–0.00251	0.00009	0.00405	0.00383	0.02334	0.00399	0.00397
	*inf*	*sq*	–0.02218	0.00073	0.00270	0.00521	0.00502	0.16545	0.03640	0.00531	0.00572	0.00608
		*lx*_(0.5)_	–0.00979	0.00410	–0.01298	0.01494	0.01325	0.19916	0.04775	0.01834	0.00769	0.00758
		*lx*(–1)	–0.05055	–0.00755	–0.00475	0.00516	0.00465	0.20085	0.03638	0.00394	0.00568	0.00599
		*lx*_(1)_	0.02942	0.01080	–0.01251	–0.00431	–0.00249	0.08867	0.02901	0.01475	0.00491	0.00552
σ	*MLE*		–0.00447	–0.00057	0.00089	0.00112	0.00054	0.00482	0.00472	0.00430	0.00414	0.00419
	*non*	*sq*	0.19462	0.08020	0.04396	0.00573	0.00016	1.01935	0.01516	0.00642	0.00380	0.00402
		*lx*_(0.5)_	0.17653	0.08104	0.07838	0.00537	–0.00001	0.11946	0.01628	0.02144	0.00384	0.00405
		*lx*_(−1)_	0.14979	0.07727	0.04334	0.00650	0.00054	0.11940	0.02539	0.00632	0.00372	0.00397
		*lx*_(1)_	–0.00724	–0.00132	0.12422	0.00106	0.00050	0.00479	0.00474	0.03138	0.00415	0.00420
	*inf*	*sq*	0.22814	0.12425	0.04490	0.01053	0.00725	0.56453	0.05027	0.00734	0.00501	0.00623
		*lx*_(0.5)_	0.49416	0.22075	0.07584	0.01463	0.00677	0.82595	0.10838	0.02046	0.00532	0.00633
		*lx*_(−1)_	0.18032	0.10940	0.00054	0.01128	0.00730	0.20176	0.06498	0.00433	0.00487	0.00616
		*lx*_(1)_	0.08097	0.05714	0.03995	0.00609	0.00810	0.14002	0.05097	0.02201	0.00574	0.00679
ξ	*MLE*		0.00756	0.00309	0.00241	0.00086	0.00521	0.00402	0.00381	0.00374	0.00376	0.00371
	*non*	*sq*	–0.04419	0.00617	0.01462	0.01428	0.01546	0.09600	0.00884	0.00553	0.00463	0.00437
		*lx*_(0.5)_	–0.06238	0.01193	–0.05911	0.02828	0.02616	0.11260	0.02057	0.02848	0.00734	0.00631
		*lx*_(−1)_	–0.03476	0.00390	0.02730	0.01440	0.01545	0.07626	0.02698	0.01041	0.00461	0.00434
		*lx*_(1)_	–0.00374	–0.00068	–0.03990	0.00035	0.00496	0.00436	0.00368	0.03538	0.00375	0.00370
	inf	*sq*	–0.43231	–0.16493	0.01595	–0.00323	0.00392	1.43579	0.12646	0.00558	0.00541	0.00545
		*lx*_(0.5)_	–0.30493	–0.13241	–0.06986	0.01102	0.01499	0.55662	0.11105	0.03305	0.00821	0.00760
		*lx*_(−1)_	–0.50315	–0.21672	0.00035	–0.00356	0.00329	1.15640	0.20561	0.00367	0.00536	0.00535
		*lx*_(1)_	–0.25142	–0.13435	–0.06515	–0.01673	–0.00599	0.20295	0.09803	0.01879	0.00446	0.00466

**Table 5:** The result of the confidence interval for the parameter estimation.

			*n* = 50	*n* = 100	*n* = 200	*n* = 500	*n* = 1000
*q* = 0.1	Fixed	LB	–0.20791	–0.13700	–0.07150	–0.00305	0.03290
		UB	0.45571	0.38540	0.32194	0.25904	0.22138
		CL	0.66363	0.52239	0.39345	0.26209	0.18848
	Discreet uniform	LB	–0.20878	–0.16003	–0.09757	–0.03483	0.00241
		UB	0.42870	0.38398	0.31964	0.26080	0.22117
		CL	0.63748	0.54401	0.41721	0.29563	0.21876
	Binomial	LB	–0.17591	–0.13309	–0.11398	–0.09637	–0.03510
		UB	0.39994	0.35606	0.33815	0.32302	0.25913
		CL	0.57586	0.48915	0.45213	0.41939	0.29422
μ=0.5	Fixed	LB	0.33756	0.40208	0.43981	0.46698	0.48111
		UB	0.69788	0.63312	0.59167	0.55838	0.54473
		CL	0.36033	0.23103	0.15187	0.09140	0.06362
	Discreet uniform	LB	0.32269	0.38922	0.42495	0.45034	0.46001
		UB	0.67642	0.61499	0.57643	0.54737	0.53050
		CL	0.35373	0.22578	0.15148	0.09703	0.07048
	Binomial	LB	0.31517	0.37861	0.40576	0.42430	0.45023
		UB	0.68452	0.61472	0.58813	0.57607	0.54562
		CL	0.36934	0.23611	0.18237	0.15177	0.09539
σ=0.5	Fixed	LB	0.41917	0.45746	0.47988	0.49989	0.50589
		UB	0.62887	0.59106	0.57092	0.55631	0.54863
		CL	0.20970	0.13359	0.09105	0.05642	0.04274
	Discreet uniform	LB	0.40335	0.43152	0.44550	0.45782	0.47306
		UB	0.59263	0.57057	0.55180	0.53426	0.52951
		CL	0.18928	0.13905	0.10630	0.07645	0.05645
	Binomial	LB	0.39208	0.43172	0.44287	0.44724	0.46419
		UB	0.59898	0.56714	0.55891	0.55125	0.53805
		CL	0.20691	0.13542	0.11604	0.10401	0.07386
ξ=0.2	Fixed	LB	–0.00664	0.08872	0.13721	0.16917	0.18557
		UB	0.46560	0.36688	0.31443	0.27681	0.25991
		CL	0.47225	0.27816	0.17722	0.10764	0.07434
	Discreet uniform	LB	0.00062	0.07711	0.12018	0.14777	0.16268
		UB	0.40913	0.32923	0.28388	0.25201	0.23990
		CL	0.40851	0.25211	0.16371	0.10424	0.07722
	Binomial	LB	–0.01622	0.06790	0.10254	0.11979	0.14885
		UB	0.43133	0.33828	0.30229	0.28237	0.25286
		CL	0.44755	0.27038	0.19975	0.16258	0.10400

From Tables 2, 3, 4, and 5

1. The MLE consistently performs well in both *Bias* and *MSE* across all sample sizes, illustrating one of the proposed PSO algorithm’s main advantages.
2. The Bayesian estimators change according to the sample size and distribution, demonstrating significant improvement as the sample size grows.
3. The *lx*_(0.5)_ estimator has the largest *MSE* and *Bias*, especially for small sample sizes.
4. The Lindley approach is especially successful with larger sample sizes, as suggested by [28]. Nevertheless, it works well with samples of 100 or more but is inappropriate for the proposed distribution when sample sizes are less than 50.
5. The result of the confidence interval demonstrates improvement with the sample size increase as a decrease of the size of the interval.

## 6 Real data

Since investigating real estate prices is important due to it’s affecting wealth distribution, economic stability, and personal finances. So, in the present paper, a data set of size 297 representing the home price data in California from 2020 until now is considered. For the data source see [29] and provided at Table 6.

**Table 6:** The real data set.

187686.8342	188317.7056	189169.5366	191018.6077	193167.8742	195486.9265	197936.0115	200492.9855
203055.8932	205486.3613	207879.9116	210222.4677	212204.1549	214163.6364	216369.1943	219111.5063
221859.4958	224355.8169	226692.9371	228944.7873	231130.4190	233174.0316	235093.8773	236889.1811
238339.7161	239822.7060	241558.5084	243900.7156	246635.5159	253180.1065	256958.8589	260776.5086
264399.3594	267948.7763	271448.8521	274541.5658	277424.9409	280479.1703	284029.2864	287971.9288
291618.7505	295468.9081	299523.9433	304028.5868	308416.4340	312644.4778	316733.0255	320965.6605
325732.8474	331170.3560	337412.4895	344599.7821	352620.9046	360821.4484	368647.6555	376059.3491
383177.6828	389435.9168	395482.2503	400863.1153	406695.1464	412825.5521	419738.1236	426462.1499
432679.9866	438628.9663	444663.4416	450725.6162	456675.9944	461503.0807	465775.9430	469335.9645
472502.2682	475518.2030	478226.0434	480916.8059	482777.8845	483468.9546	483475.0609	482439.0074
481258.3361	479999.4396	478907.2086	478005.7119	476948.1764	475435.2246	473495.6873	470470.4004
466986.3606	462527.4081	457877.7438	452652.2220	447168.4820	441656.1350	435747.0121	429862.8846
422917.7863	414621.4368	404896.4137	394544.7294	384492.0353	374310.7423	364509.0873	355453.5520
347622.7915	340031.0702	332747.0293	325938.0673	320979.5349	316872.6546	313013.5797	309274.0082
306005.8766	303582.2358	301556.5902	299969.9176	299124.1092	299957.3709	301864.6029	303801.6852
304610.9382	305644.9033	306932.7221	308350.6835	308285.7308	307148.4770	305205.8801	303223.8202
301097.3364	299213.7398	297872.1271	296827.4453	295826.5874	294352.1849	292782.8317	290425.0487
288527.3871	286874.9231	285072.5041	282920.9726	280623.8657	279489.6153	278750.3796	278369.3156
277815.5125	277193.0813	276768.0442	276961.4488	278056.0615	279643.4480	282153.4359	285543.1346
289868.9139	294605.6162	299647.0704	304422.5972	308648.7275	312278.9999	316552.4719	322390.0959
329454.1342	336530.3735	343298.6289	349931.4467	356482.3765	361977.3292	366403.6066	369607.2530
372255.8784	374664.6530	377602.2942	380737.0625	383318.5964	385016.0004	386504.2009	388961.1591
392077.8866	395570.7508	398795.8141	401663.1416	404086.5822	406070.1354	407989.9860	410160.7676
412385.3681	414138.3455	415313.1620	416318.0286	417556.7026	418590.5136	419533.4667	422657.4409
427912.8469	433882.7061	438393.3644	441385.4760	444029.5279	444996.5575	444692.1484	443230.8109
442898.6128	443952.8783	446497.6616	451311.7572	457646.8320	464527.0834	470146.0013	474265.7097
477796.4285	479515.3408	481001.0254	482565.3023	485967.0300	489739.2045	494172.6047	499178.8081
504700.0779	509733.0955	513872.8811	517660.4111	520743.3764	523548.1668	526557.2370	529952.2831
533563.5770	536117.3334	537613.6398	537146.9730	535542.3357	533724.2869	532680.8200	532056.1442
531670.9291	532075.7347	533768.7461	536356.5770	539407.0740	542728.4508	546170.6719	548982.1261
551243.1015	553421.4450	555432.9076	555686.9550	554483.4320	554328.1230	557564.7191	564750.0842
573976.5901	584720.3715	595436.9155	604335.7805	611949.5190	618846.9364	627876.5723	638689.0126
650297.9802	660790.4137	668049.6582	672972.0653	677366.5178	683160.6964	690303.6065	700265.1966
713048.1115	728462.2517	742479.5383	753045.5170	758663.2687	758970.2853	754338.9832	746931.8920
740263.6993	735023.4323	730531.5843	723543.2201	716358.6239	711073.6556	710491.4903	713456.5982
718649.1184	725648.6790	733568.9728	741137.6436	747291.8822	751695.9037	754499.8326	755031.2293
754878.4575	756360.2570	760219.9022	764707.1623	767178.6187	769174.4409	770943.0607	773239.1176

**Fig 4:**
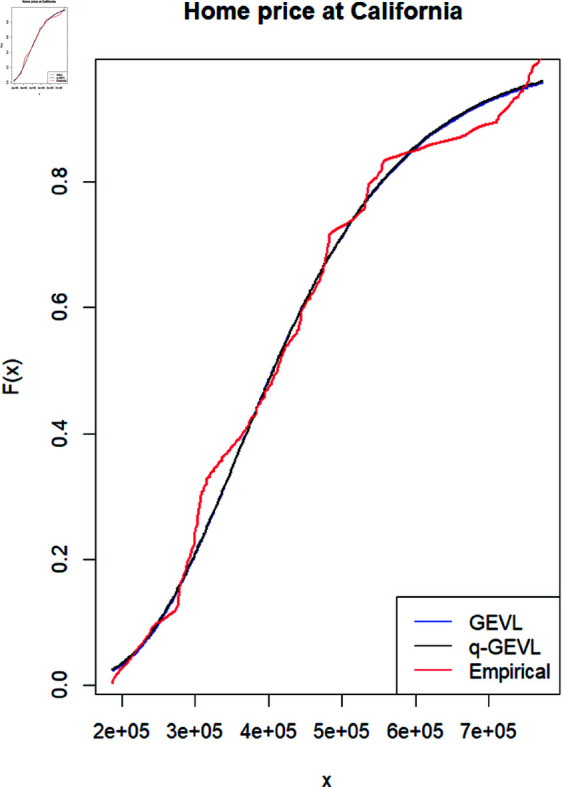
The CDF and Empirical Distribution for both GEVL and q-GEVL for home price in California.

These data set is fitted to both GEVL and q−GEVL and the results obtained into Table 7 and Fig 4 show that both *GEVL* and q−GEVL are giving a good fit for this data using different types of fitting measures.

**Table 7:** The different fitting measures for home price in California.

Distribution	MLE	${KS}_{0.05}=0.0947$	-log(L)	BIC	AIC
GEVL	(3.5842e+05, 1.2933e+05, -1.2256e-02)	7.6278e-02	3.9411e+03	7.8993e+03	7.8882e+03
q-GEVL	(0.01, 3.5912e+05, 1.2945e+05, 0.1256e-02)	7.5320e-02	3.9413e+03	7.9054e+03	7.8906e+03

More over, a summary of statistics and statistical properties of this data set are provided in both Tables 8 and 9.

**Fig 5:**
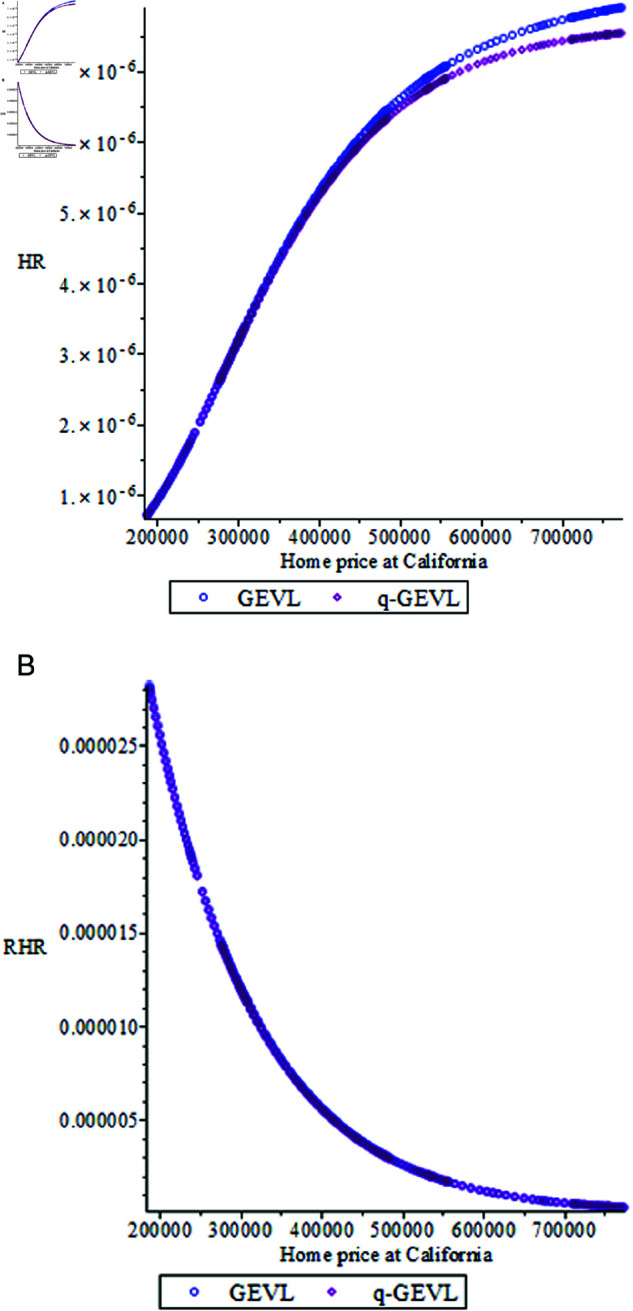
The HR and RHR for home price of real state at California.

From Tables 8 and 9, and Fig 5:

- Both distributions have positive skewness, indicating a slight right tail (more extreme high values than low values).
- Both distributions have kurtosis values greater than 1. Then both distributions exhibit a slightly heavier tail than a normal distribution (platykurtic).
- The q−GEVL model predicts higher return levels compared to *GEVL*, especially for larger return periods (20,30) years which. This is indicated to predict more extreme home price values over time, which is compatible with the results in Table 1.

**Table 8:** Summary of the Statistics for Home Price in California.

Quartiles(25%,50%,75%)	Range	Max	Min	Mean	Median	Variance	Standard Deviation
(301787.6, 412605.5, 524300.4)	585555.3	773239.1	187686.8	429751.5	412605.5	24392554126	156181.2

**Table 9:** The statistical properties for California.

	SK	KR	(t=10,20,30)
GEVL	0.1176	1.2786	( 6.454331*105, 5.506350*105, 4.909114*105)
q-GEVL	0.1136	1.2739	(6.507737*105, 7.442694*105, 7.981273*105)

## 7 Conclusion

In this paper, the properties of *q*-extended extreme value distribution with linear normalization q−GEVL are displayed showing that q−GEVL is particularly useful for scenarios where modeling extreme lower-end behavior as financial risk and environmental extremes are critical. Then the Particle Swarm Optimization (PSO) for estimating the MLE of q−GEVL parameters is used. Both MLE and BE under type-II progressive censoring schemes (fixed, binomial, and discrete uniform random removal distributions) are conducted for q−GEVL parameters. Both point and interval estimation are considered for parameters MLE. While Lindley’s approximation was used in Bayesian estimation for both informative and non-informative priors, under the square error and LINEX loss functions. Moreover, simulation is presented showing that MLE consistently produces outstanding results in both *Bias* and *MSE* for different sample sizes, highlighting the strengths of the suggested PSO algorithm. Contrary the performance of Bayesian estimators fluctuates according to sample size and distribution, with substantial improvements as sample sizes increase. The *lx*_(0.5)_ estimator provides the highest *MSE* and Bias, especially if the sample size is small. The Lindley method’s performs well for sample sizes of 100 or more but fails to perform for the suggested distribution q−GEVL when the sample size is less than 50. Then an example of real home price in California is considered as one of the applications on q−GEVL showing that q−GEVL gives to predict more extreme home price values over time, which is compatible with the results in Table 1.

## References

[1] Gilli M, këllezi E. An application of extreme value theory for measuring financial risk. Comput Econ. 2006;27(2–3):207–28. doi: 10.1007/s10614-006-9025-7

[2] Fisher RA, Tippett LHC. Limiting forms of the frequency distribution of the largest or smallest member of a sample. Math Proc Camb Phil Soc. 1928;24(2):180–90. doi: 10.1017/s0305004100015681

[3] Bali TG. The generalized extreme value distribution. Econ Lett. 2003;79(3):423–7. doi: 10.1016/s0165-1765(03)00035-1

[4] Bertin E, Clusel M. Generalized extreme value statistics and sum of correlated variables. J Phys A: Math Gen. 2006;39(24):7607–19. doi: 10.1088/0305-4470/39/24/001

[5] Hosking JRM, Wallis JR, Wood EF. Estimation of the generalized extreme-value distribution by the method of probability-weighted moments. Technometrics. 1985;27(3):251–61. doi: 10.1080/00401706.1985.10488049

[6] Bleed SO, Attwa RA-E, Ali RFM, Radwan T. On alpha power transformation generalized pareto distribution and some properties. J Appl Math. 2024;2024(1):6270350. doi: 10.1155/2024/6270350

[7] Attwa RA-E, Radwan T, Zaid EOA. Bivariate q-extended Weibull morgenstern family and correlation coefficient formulas for some of its sub-models. MATH. 2023;8(11):25325–42. doi: 10.3934/math.20231292

[8] Attwa R, Zaid E. Record values from the Gumbel and q-Gumbel distributions with applications. Thailand Stat. 2024;22(4):750–68.

[9] Dey S, Sharma VK, Mesfioui M. A new extension of weibull distribution with application to lifetime data. Ann Data Sci. 2017;4(1):31–61. doi: 10.1007/s40745-016-0094-8

[10] Mudholkar GS, Srivastava DK. Exponentiated Weibull family for analyzing bathtub failure-rate data. IEEE Trans Rel. 1993;42(2):299–302. doi: 10.1109/24.229504

[11] Marshall A, Olkin I. A new method for adding a parameter to a family of distributions with application to the exponential and Weibull families. Biometrika. 1997;84(3):641–52.

[12] Eugene N, Lee C, Famoye F. Beta-normal distribution and its applications. Commun Statist Theory Methods. 2002;31(4):497–512. doi: 10.1081/sta-120003130

[13] Provost S, Saboor A, Cordeiro G, Mansoor M. On the q-generalized extreme value distribution. Revstat-Statist J. 2018;16(1):45–70.

[14] Zaid EOA, Attwa RAE-W, Radwan T. Some measures information for generalized and q-generalized extreme values and its properties. Fractals. 2022;30(10):2240246. doi: 10.1142/s0218348x22402460

[15] Attwa RAE-W, Radwan T. Applying generalized Type-II hybrid censored samples on generalized and q-generalized extreme value distributions under linear normalization. Symmetry. 2023;15(10):1869. doi: 10.3390/sym15101869

[16] Nair SS, Jayakumar K. Generalized q -logistic distribution. Commun Statist- Simulat Comput. 2022;53(8):3771–87. doi: 10.1080/03610918.2022.2112055

[17] Mathai A, Provost S. The q-extended inverse Gaussian distribution. J Probab Stat Sci. 2011;9:1–20.

[18] Budini AA. Extended q-Gaussian and q-exponential distributions from gamma random variables. Phys Rev E Stat Nonlin Soft Matter Phys. 2015;91(5):052113. doi: 10.1103/PhysRevE.91.052113 26066125

[19] Afify AZ, Zayed M, Ahsanullah M. The extended exponential distribution and its applications. JSTA. 2018;17(2):213. doi: 10.2991/jsta.2018.17.2.3

[20] Balakrishnan N, Aggarwala R. Progressive censoring: theory, methods, and applications. New York: Springer; 2000.

[21] Yao H, Gui W. Inference on exponentiated Rayleigh distribution with constant stress partially accelerated life tests under progressive type-II censoring. J Appl Stat. 2024:1–29. 10.1080/02664763.2024.2373930 PMC11800349 39926178

[22] Prakash A, Maurya RK, Alsadat N, Obulezi OJ. Parameter estimation for reduced Type-I Heavy-Tailed Weibull distribution under progressive Type-II censoring scheme. Alexandria Eng J. 2024;109:935–49. doi: 10.1016/j.aej.2024.09.029

[23] Maiti K, Kayal S. Estimation of parameters and reliability characteristics for a generalized Rayleigh distribution under progressive type-II censored sample. Commun Statist - Simulat Comput. 2019;50(11):3669–98. doi: 10.1080/03610918.2019.1630431

[24] Sharma A, Sharma A, Pandey JK, Ram M. Swarm intelligence: foundation, principles, and engineering applications. CRC Press; 2022.

[25] Attwa RAE-W, Sadk SW, Aljohani HM. Investigation the generalized extreme value under liner distribution parameters for progressive type-II censoring by using optimization algorithms. MATH. 2024;9(6):15276–302. doi: 10.3934/math.2024742

[26] Particle swarm optimization with R. [cited 2024 Oct 19]. 2022. Available from: https://reintech.io/blog/particle-swarm-optimization-with-r

[27] Attwa RAE-W, Sadk SW, Radwan T. Estimation of Marshall–Olkin extended generalized extreme value distribution parameters under progressive Type-II censoring by using a genetic algorithm. Symmetry. 2024;16(6):669. doi: 10.3390/sym16060669

[28] Lindley DV. Approximate Bayesian methods. Trabajos de Estadistica Y de Investigacion Operativa. 1980;31(1):223–45. doi: 10.1007/bf02888353

[29] Zillow. Zillow research data. [cited 2024 Nov 01]. 2024. Available from: https://www.zillow.com/research/data/

